# Supplementary material for: Validating a model of architectural hazard visibility with low-vision observers
Source: PLoS One. 2021 Nov 22;16(11):e0260267. doi: 10.1371/journal.pone.0260267 (PMC8608317; doi:10.1371/journal.pone.0260267)
Supplement: S1 Appendix — (DOCX) [file pone.0260267.s001.docx]

# S1 Appendix: Formula for HVS derivation

In the method for estimating visibility of architectural features, we evaluate a feature’s visibility by comparing the luminance boundary captured from the low-vision-simulation filtered image and the geometrical boundary derived from the 3D map. For each pixel on the geometry boundary, we evaluate its local visibility through the following formula:

$h\left( i,j \right)=1-e^{-\left( \frac{d_{\mathrm{angle}}}{2\theta_{h}} \right)^{2}}$

where:

$h\left( i,j \right)$ = the magnitude of hazard. $i$ and $j$ are the coordinates of the pixel being evaluated in the image;

$d_{\mathrm{angle}}$ = pixel separation in units of visual angle between the evaluated pixel and its closest neighbor on the luminance boundary;

$\theta_{h}$ = scaling parameter, which is set to be 0.75 in the stimuli used in this paper.
