# Supplementary material for: Validating a model of architectural hazard visibility with low-vision observers
Source: PLoS One. 2021 Nov 22;16(11):e0260267. doi: 10.1371/journal.pone.0260267 (PMC8608317; doi:10.1371/journal.pone.0260267)
Supplement: S2 Appendix — (DOCX) [file pone.0260267.s002.docx]

# S2 Appendix: Experiment 1 Severe blur group high-HVS trials confusion matrix

| True\response | Big Up | Big Down | Small Up | Small Down | Flat | Sum |
| --- | --- | --- | --- | --- | --- | --- |
| Big up | 79 | 27 | 50 | 4 | 2 | 162 |
| Big Down | 0 | 0 | 0 | 0 | 0 | 0 |
| Small Up | 0 | 0 | 0 | 0 | 0 | 0 |
| Small Down | 0 | 0 | 0 | 0 | 0 | 0 |
| Flat | 5 | 8 | 5 | 2 | 18 | 38 |
| Sum | 84 | 35 | 55 | 6 | 20 | 200 |

*Confusion matrix of above-0.8-HVS trials accumulated from seven subjects in the severe blur group.*
