# Supplementary material for: Validating a model of architectural hazard visibility with low-vision observers
Source: PLoS One. 2021 Nov 22;16(11):e0260267. doi: 10.1371/journal.pone.0260267 (PMC8608317; doi:10.1371/journal.pone.0260267)
Supplement: S3 Appendix — (DOCX) [file pone.0260267.s003.docx]

# S3 Appendix: Individual regression models of low-vision subjects fitted with alternative (central only) ROI

*Table. Comparison of slopes, null and residual deviance, and reduced deviance ratio of ten low-vision individual regression model fitted with complete ROI and central ROI. Subject No. 2, 3, 4, 6, 9 had fitted regression models improved by changing ROI definition from complete ROI to central ROI.*

|  | VA | CS | Complete ROI Slope | Complete ROI Null Dev | Complete ROI Resid. Dev | Complete ROI Reduced Deviance Ratio | Central ROI Slope | Central ROI Null Dev | Central ROI Resid. Dev | Central ROI Reduced Deviance Ratio |
| --- | --- | --- | --- | --- | --- | --- | --- | --- | --- | --- |
| LV1 | 0.8 | 1.65 | 6.45 | 70.814 | 52.825 | 25% | 4.19 | 68 | 56 | 18% |
| LV2 | 1.28 | 0.6 | 1.57 | 265.96 | 255.75 | 4% | 2.26 | 217 | 198 | 9% |
| LV3 | 1.14 | 0.3 | 3.23 | 337.3 | 276.59 | 18% | 4.11 | 302 | 225 | 25% |
| LV4 | 1.16 | 1.05 | 5.6 | 265.96 | 178.18 | 33% | 7.61 | 242 | 142 | 41% |
| LV5 | 1.5 | 0.3 | 5.56 | 338.79 | 267.44 | 21% | 6.03 | 304 | 234 | 23% |
| LV6 | 1.36 | 0.8 | 3.79 | 326.71 | 266.45 | 18% | 3.92 | 288 | 219 | 24% |
| LV7 | 1.44 | 0.2 | 2.27 | 346.51 | 327.5 | 5% | 3.33 | 321 | 296 | 8% |
| LV8 | 1.54 | 0.65 | 3.04 | 344.27 | 302.38 | 12% | 2.96 | 302 | 257 | 15% |
| LV9 | 1.66 | 0.57 | 2.53 | 324.34 | 302.1 | 7% | 2.63 | 287 | 254 | 11% |
| LV10 | 1.02 | 1.55 | 11.03 | 124.22 | 72.188 | 42% | 22.92 | 119 | 65 | 45% |
