# Supplementary material for: Validating a model of architectural hazard visibility with low-vision observers
Source: PLoS One. 2021 Nov 22;16(11):e0260267. doi: 10.1371/journal.pone.0260267 (PMC8608317; doi:10.1371/journal.pone.0260267)
Supplement: S1 File — (PDF) [file pone.0260267.s004.pdf]

Validating a Model of Architectural Hazard Visibility with Low-Vision Observers - Supplementary File

This document contains the supplementary data for the paper "Validating a Model of Architectural Hazard Visibility with Low-Vision Observers".

```
### Prepare libraries
install.packages("MuMIn",repos = "http://cran.us.r-project.org")
```

```
## Installing package into 'C:/Users/6174l/Documents/R/win-library/4.0'
## (as 'lib' is unspecified)
```

```
## package 'MuMIn' successfully unpacked and MD5 sums checked
##
## The downloaded binary packages are in
##  C:\Users\6174l\AppData\Local\Temp\Rtmpieh2h1\downloaded_packages
```

```
install.packages("DescTools",repos = "http://cran.us.r-project.org")
```

```
## Installing package into 'C:/Users/6174l/Documents/R/win-library/4.0'
## (as 'lib' is unspecified)
```

```
## package 'DescTools' successfully unpacked and MD5 sums checked
##
## The downloaded binary packages are in
##  C:\Users\6174l\AppData\Local\Temp\Rtmpieh2h1\downloaded_packages
```

```
install.packages("powerMediation",repos = "http://cran.us.r-project.org")
```

```
## Installing package into 'C:/Users/6174l/Documents/R/win-library/4.0'
## (as 'lib' is unspecified)
```

```
## package 'powerMediation' successfully unpacked and MD5 sums checked
##
## The downloaded binary packages are in
##  C:\Users\6174l\AppData\Local\Temp\Rtmpieh2h1\downloaded_packages
```

```
install.packages("lme4",repos = "http://cran.us.r-project.org")
```

```
## Installing package into 'C:/Users/6174l/Documents/R/win-library/4.0'
## (as 'lib' is unspecified)
```

```
## package 'lme4' successfully unpacked and MD5 sums checked
##
## The downloaded binary packages are in
##  C:\Users\61741\AppData\Local\Temp\Rtmpieh2h1\downloaded_packages
```

```
install.packages('gap',repos = "http://cran.us.r-project.org")
```

```
## Installing package into 'C:/Users/61741/Documents/R/win-library/4.0'
## (as 'lib' is unspecified)
```

```
## package 'gap' successfully unpacked and MD5 sums checked
##
## The downloaded binary packages are in
##  C:\Users\61741\AppData\Local\Temp\Rtmpieh2h1\downloaded_packages
```

```
library(MuMIn)
library(DescTools)
library(powerMediation)
library(lme4)
```

```
## Loading required package: Matrix
```

```
library(gap)
```

```
## gap version 1.2.2
```

```
### Read Data

# Sim Low Vision - UoM Generated HVS Score File
data.sim = read.csv("SubjCSV_UoM.csv", header = F)
colnames(data.sim) <- c("x","y","id","geom","light","view")
data.sim$id <- as.factor(data.sim$id)
data.sim$y <- as.factor(2 - data.sim$y)
# Severe condition
# data.sev = read.csv("SLVCSV_UoM_Sev.csv", header = F)
data.sev = read.csv("SLVCSV_UoM_Sev.csv", header = F)
colnames(data.sev) <- c("x","y","id","geom","light","view")
data.sev$id <- as.factor(data.sev$id)
data.sev$y <- as.factor(2 - data.sev$y)
# Moderate condition
data.mod = read.csv("SLVCSV_UoM_Mod.csv", header = F)
# data.sev = read.csv("SubjCSV_UoM_Sev.csv", header = F)
colnames(data.mod) <- c("x","y","id","geom","light","view")
data.mod$id <- as.factor(data.mod$id)
data.mod$y <- as.factor(2 - data.mod$y)
# Real low-vision subjects
data.RLV = read.csv("RLVCSV_UoM.csv", header = F)
colnames(data.RLV) <- c("x","y","id","geom","light","view")
data.RLV$id <- as.factor(data.RLV$id)
data.RLV$y <- as.factor(2 - data.RLV$y)
#Real low-vision subjects with Central ROI
```

```r
data.RLVC = read.csv("RLVCSV_ROI_Central.csv", header = F)
colnames(data.RLVC) <- c("x","y","id","geom","light","view")
data.RLVC$id <- as.factor(data.RLVC$id)
data.RLVC$y <- as.factor(2 - data.RLVC$y)

RLV.VA = c(0.8, 1.28, 1.14, 1.16, 1.5, 1.36, 1.44, 1.54, 1.66, 1.02)
RLV.CS = c(1.65, 0.6, 0.3, 1.05, 0.3, 0.8, 0.2, 0.65, 0.57, 1.55)
```

Accumulated Data

Experiment 1 - Overall

```r
model.sim <- glmer(y~x + (1|id), family = "binomial", data = data.sim)
summary(model.sim)
```

```
## Generalized linear mixed model fit by maximum likelihood (Laplace
##   Approximation) [glmerMod]
##  Family: binomial  ( logit )
## Formula: y ~ x + (1 | id)
##    Data: data.sim
##
##      AIC      BIC   logLik deviance df.resid
##   4127.0   4145.5  -2060.5   4121.0     3497
##
## Scaled residuals:
##     Min      1Q  Median      3Q     Max
## -2.9544 -0.7711 -0.3436  0.7735  3.0435
##
## Random effects:
##  Groups Name        Variance Std.Dev.
##  id     (Intercept) 0.4306   0.6562
## Number of obs: 3500, groups:  id, 14
##
## Fixed effects:
##             Estimate Std. Error z value Pr(>|z|)
## (Intercept)  -1.0996     0.1891  -5.816 6.03e-09 ***
## x             2.3485     0.1316  17.852  < 2e-16 ***
## ---
## Signif. codes:  0 '***' 0.001 '**' 0.01 '*' 0.05 '.' 0.1 ' ' 1
##
## Correlation of Fixed Effects:
##   (Intr)
## x -0.315
```

```r
anova(model.sim, test = 'Chi')
```

```
## Warning in anova.merMod(model.sim, test = "Chi"): additional arguments ignored:
## 'test'

## Analysis of Variance Table
##   npar Sum Sq Mean Sq F value
## x    1 319.35  319.35  319.35
```

Experiment 1 - Moderate Blur

```
model.moderate <- glmer(y~x + (1|id), family = "binomial", data = data.mod)
summary(model.moderate)
```

```
## Generalized linear mixed model fit by maximum likelihood (Laplace
##   Approximation) [glmerMod]
##  Family: binomial  ( logit )
## Formula: y ~ x + (1 | id)
##    Data: data.mod
##
##      AIC      BIC   logLik deviance df.resid
##   1930.7   1947.1   -962.4   1924.7     1747
##
## Scaled residuals:
##     Min      1Q  Median      3Q     Max
## -3.5380 -0.8224  0.3575  0.6897  2.0245
##
## Random effects:
##  Groups Name        Variance Std.Dev.
##  id     (Intercept) 0.1678   0.4096
## Number of obs: 1750, groups:  id, 7
##
## Fixed effects:
##             Estimate Std. Error z value Pr(>|z|)
## (Intercept)  -0.8595     0.1861  -4.618 3.88e-06 ***
## x             3.0287     0.1919  15.782  < 2e-16 ***
## ---
## Signif. codes:  0 '***' 0.001 '**' 0.01 '*' 0.05 '.' 0.1 ' ' 1
##
## Correlation of Fixed Effects:
##   (Intr)
## x -0.467
```

```
anova(model.moderate, test = 'Chi')
```

```
## Warning in anova.merMod(model.moderate, test = "Chi"): additional arguments
## ignored: 'test'
```

```
## Analysis of Variance Table
##   npar Sum Sq Mean Sq F value
## x    1  251.6   251.6   251.6
```

Experiment 1 - Severe Blur

```
model.severe <- glmer(y~x + (1|id), family = "binomial", data = data.sev)
summary(model.severe)
```

```
## Generalized linear mixed model fit by maximum likelihood (Laplace
```

```
##   Approximation) [glmerMod]
##  Family: binomial  ( logit )
## Formula: y ~ x + (1 | id)
##    Data: data.sev
##
##      AIC      BIC   logLik deviance df.resid
##   2155.0   2171.4  -1074.5   2149.0     1747
##
## Scaled residuals:
##     Min      1Q  Median      3Q     Max
## -1.2285 -0.7127 -0.5759  1.0616  2.4350
##
## Random effects:
##  Groups Name        Variance Std.Dev.
##  id     (Intercept) 0.08777  0.2963
## Number of obs: 1750, groups:  id, 7
##
## Fixed effects:
##             Estimate Std. Error z value Pr(>|z|)
## (Intercept)  -1.3125     0.1466  -8.953  < 2e-16 ***
## x             1.5441     0.1896   8.142 3.89e-16 ***
## ---
## Signif. codes:  0 '***' 0.001 '**' 0.01 '*' 0.05 '.' 0.1 ' ' 1
##
## Correlation of Fixed Effects:
##   (Intr)
## x -0.538
```

```r
anova(model.severe, test = 'Chi')
```

```
## Warning in anova.merMod(model.severe, test = "Chi"): additional arguments
## ignored: 'test'
```

```
## Analysis of Variance Table
##   npar Sum Sq Mean Sq F value
## x    1 66.644  66.644  66.644
```

Experiment 1 - Comparing blur conditions

```r
sim.compare = chow.test(data.sev$y,data.sev$x,data.mod$y, data.mod$x)
sim.compare
```

```
##      F value        d.f.1        d.f.2      P value
## 1.259795e+02 2.000000e+00 3.496000e+03 1.477292e-53
```

Experiment 2

```r
model.RLV <- glmer(y~x + (1|id), family = "binomial", data = data.RLV)
summary(model.RLV)
```

```
## Generalized linear mixed model fit by maximum likelihood (Laplace
##   Approximation) [glmerMod]
##  Family: binomial  ( logit )
## Formula: y ~ x + (1 | id)
##    Data: data.RLV
##
##      AIC      BIC   logLik deviance df.resid
##   2403.4   2420.8  -1198.7   2397.4     2497
##
## Scaled residuals:
##     Min      1Q  Median      3Q     Max
## -9.7123 -0.6876  0.2225  0.5802  2.5711
##
## Random effects:
##  Groups Name        Variance Std.Dev.
##  id     (Intercept) 0.8735   0.9346
## Number of obs: 2500, groups:  id, 10
##
## Fixed effects:
##             Estimate Std. Error z value Pr(>|z|)
## (Intercept)  -0.6194     0.3093  -2.002   0.0452 *
## x             3.4595     0.1971  17.548   <2e-16 ***
## ---
## Signif. codes:  0 '***' 0.001 '**' 0.01 '*' 0.05 '.' 0.1 ' ' 1
##
## Correlation of Fixed Effects:
##   (Intr)
## x -0.224
```

```
anova(model.RLV, test = 'Chi')
```

```
## Warning in anova.merMod(model.RLV, test = "Chi"): additional arguments ignored:
## 'test'
```

```
## Analysis of Variance Table
##   npar Sum Sq Mean Sq F value
## x    1  307.5   307.5   307.5
```

Individual Data

Experiment 1

```
p = length(levels(data.mod$id))
Mod.collect <- list()
Mod.OR.collect <- list()
Mod.p.OR.collect <- list()
Mod.confint.collect <- list()

library(MuMIn)
library(DescTools)
for (i in 1:p){
```

```r
  lev = levels(data.mod$id)[i]
  sub1 <- data.mod[data.mod$id==lev,]
  model = glm(y~x,family = "binomial",data = sub1)
  Mod.collect[[i]] = model
  Mod.OR.collect[[i]] <- exp(model$coefficients[2])
  Mod.p.OR.collect[[i]] <- summary(model)$coefficients[2,4]
  flush.console()
  Mod.confint.collect[[i]] <- confint.default(model)
  cat("Subject id: ", levels(data.mod$id)[i], ", Logistic Regression Model Summary")
  print(summary(model))
  cat("Subject id: ", levels(data.mod$id)[i])
  print(summary(model)$coefficients)
  cat("Subject id: ", levels(data.mod$id)[i], ", Null model comparison (Chi)")
  print(anova(model, test = 'Chi'))
}
```

```
## Subject id:  10 , Logistic Regression Model Summary
## Call:
## glm(formula = y ~ x, family = "binomial", data = sub1)
##
## Deviance Residuals:
##     Min       1Q   Median       3Q      Max
## -2.1930  -1.0538   0.4663   0.9088   1.5074
##
## Coefficients:
##             Estimate Std. Error z value Pr(>|z|)
## (Intercept)  -0.8126     0.2734  -2.972  0.00295 **
## x             3.1766     0.5292   6.002 1.94e-09 ***
## ---
## Signif. codes:  0 '***' 0.001 '**' 0.01 '*' 0.05 '.' 0.1 ' ' 1
##
## (Dispersion parameter for binomial family taken to be 1)
##
##     Null deviance: 313.43  on 249  degrees of freedom
## Residual deviance: 267.82  on 248  degrees of freedom
## AIC: 271.82
##
## Number of Fisher Scoring iterations: 4
##
## Subject id:  10               Estimate Std. Error   z value     Pr(>|z|)
## (Intercept) -0.8125526  0.2733643 -2.972417 2.954655e-03
## x            3.1766117  0.5292178  6.002466 1.943429e-09
## Subject id:  10 , Null model comparison (Chi)Analysis of Deviance Table
##
## Model: binomial, link: logit
##
## Response: y
##
## Terms added sequentially (first to last)
##
##
##      Df Deviance Resid. Df Resid. Dev  Pr(>Chi)
## NULL                   249     313.44
```

```
## x     1   45.618       248     267.82 1.437e-11 ***
## ---
## Signif. codes:  0 '***' 0.001 '**' 0.01 '*' 0.05 '.' 0.1 ' ' 1
## Subject id:  11 , Logistic Regression Model Summary
## Call:
## glm(formula = y ~ x, family = "binomial", data = sub1)
##
## Deviance Residuals:
##     Min       1Q   Median       3Q      Max
## -2.2651  -1.0742   0.4219   0.9118   1.3361
##
## Coefficients:
##             Estimate Std. Error z value Pr(>|z|)
## (Intercept)  -0.3744     0.2514  -1.489    0.136
## x             2.9301     0.5373   5.453 4.94e-08 ***
## ---
## Signif. codes:  0 '***' 0.001 '**' 0.01 '*' 0.05 '.' 0.1 ' ' 1
##
## (Dispersion parameter for binomial family taken to be 1)
##
##     Null deviance: 298.35  on 249  degrees of freedom
## Residual deviance: 259.94  on 248  degrees of freedom
## AIC: 263.94
##
## Number of Fisher Scoring iterations: 4
##
## Subject id:  11              Estimate Std. Error   z value     Pr(>|z|)
## (Intercept) -0.3744339  0.2514234 -1.489256 1.364198e-01
## x            2.9301497  0.5373048  5.453422 4.940969e-08
## Subject id:  11 , Null model comparison (Chi)Analysis of Deviance Table
##
## Model: binomial, link: logit
##
## Response: y
##
## Terms added sequentially (first to last)
##
##
##      Df Deviance Resid. Df Resid. Dev  Pr(>Chi)
## NULL                   249     298.35
## x     1    38.41       248     259.94 5.735e-10 ***
## ---
## Signif. codes:  0 '***' 0.001 '**' 0.01 '*' 0.05 '.' 0.1 ' ' 1
## Subject id:  12 , Logistic Regression Model Summary
## Call:
## glm(formula = y ~ x, family = "binomial", data = sub1)
##
## Deviance Residuals:
##     Min       1Q   Median       3Q      Max
## -1.8147  -0.9864   0.6584   0.8545   1.7259
##
## Coefficients:
##             Estimate Std. Error z value Pr(>|z|)
## (Intercept)  -1.3034     0.2832  -4.603 4.17e-06 ***
```

```
## x             2.7861     0.4440   6.274 3.51e-10 ***
## ---
## Signif. codes:  0 '***' 0.001 '**' 0.01 '*' 0.05 '.' 0.1 ' ' 1
##
## (Dispersion parameter for binomial family taken to be 1)
##
##     Null deviance: 341.37  on 249  degrees of freedom
## Residual deviance: 295.82  on 248  degrees of freedom
## AIC: 299.82
##
## Number of Fisher Scoring iterations: 4
##
## Subject id:  12             Estimate Std. Error   z value     Pr(>|z|)
## (Intercept) -1.303376  0.2831824 -4.602603 4.172434e-06
## x            2.786063  0.4440306  6.274483 3.507975e-10
## Subject id:  12 , Null model comparison (Chi)Analysis of Deviance Table
##
## Model: binomial, link: logit
##
## Response: y
##
## Terms added sequentially (first to last)
##
##
##      Df Deviance Resid. Df Resid. Dev  Pr(>Chi)
## NULL                   249     341.37
## x     1   45.548       248     295.82 1.489e-11 ***
## ---
## Signif. codes:  0 '***' 0.001 '**' 0.01 '*' 0.05 '.' 0.1 ' ' 1
## Subject id:  13 , Logistic Regression Model Summary
## Call:
## glm(formula = y ~ x, family = "binomial", data = sub1)
##
## Deviance Residuals:
##     Min       1Q   Median       3Q      Max
## -2.1720  -0.9813   0.4592   0.8360   1.4924
##
## Coefficients:
##             Estimate Std. Error z value Pr(>|z|)
## (Intercept)  -1.0861     0.2830  -3.838 0.000124 ***
## x             3.4959     0.5363   6.519 7.09e-11 ***
## ---
## Signif. codes:  0 '***' 0.001 '**' 0.01 '*' 0.05 '.' 0.1 ' ' 1
##
## (Dispersion parameter for binomial family taken to be 1)
##
##     Null deviance: 319.17  on 249  degrees of freedom
## Residual deviance: 263.99  on 248  degrees of freedom
## AIC: 267.99
##
## Number of Fisher Scoring iterations: 4
##
## Subject id:  13             Estimate Std. Error   z value     Pr(>|z|)
## (Intercept) -1.086103  0.2829524 -3.838466 1.238053e-04
```

```
## x            3.495860  0.5362739  6.518795 7.087433e-11
## Subject id:  13 , Null model comparison (Chi)Analysis of Deviance Table
##
## Model: binomial, link: logit
##
## Response: y
##
## Terms added sequentially (first to last)
##
##
##      Df Deviance Resid. Df Resid. Dev  Pr(>Chi)
## NULL                   249     319.17
## x     1   55.182       248     263.99 1.099e-13 ***
## ---
## Signif. codes:  0 '***' 0.001 '**' 0.01 '*' 0.05 '.' 0.1 ' ' 1
## Subject id:  16 , Logistic Regression Model Summary
## Call:
## glm(formula = y ~ x, family = "binomial", data = sub1)
##
## Deviance Residuals:
##     Min       1Q   Median       3Q      Max
## -2.6501  -0.7933   0.2794   0.6980   1.7344
##
## Coefficients:
##             Estimate Std. Error z value Pr(>|z|)
## (Intercept)  -1.7576     0.3295  -5.335 9.57e-08 ***
## x             5.2600     0.7157   7.349 1.99e-13 ***
## ---
## Signif. codes:  0 '***' 0.001 '**' 0.01 '*' 0.05 '.' 0.1 ' ' 1
##
## (Dispersion parameter for binomial family taken to be 1)
##
##     Null deviance: 310.35  on 249  degrees of freedom
## Residual deviance: 214.96  on 248  degrees of freedom
## AIC: 218.96
##
## Number of Fisher Scoring iterations: 5
##
## Subject id:  16             Estimate Std. Error   z value     Pr(>|z|)
## (Intercept) -1.757641  0.3294725 -5.334714 9.569517e-08
## x            5.259950  0.7157164  7.349211 1.993808e-13
## Subject id:  16 , Null model comparison (Chi)Analysis of Deviance Table
##
## Model: binomial, link: logit
##
## Response: y
##
## Terms added sequentially (first to last)
##
##
##      Df Deviance Resid. Df Resid. Dev  Pr(>Chi)
## NULL                   249     310.35
## x     1   95.389       248     214.96 < 2.2e-16 ***
## ---
```

```
## Signif. codes:  0 '***' 0.001 '**' 0.01 '*' 0.05 '.' 0.1 ' ' 1
## Subject id:  17 , Logistic Regression Model Summary
## Call:
## glm(formula = y ~ x, family = "binomial", data = sub1)
##
## Deviance Residuals:
##     Min       1Q   Median       3Q      Max
## -1.6295  -1.0378   0.7761   1.0494   1.5991
##
## Coefficients:
##             Estimate Std. Error z value Pr(>|z|)
## (Intercept)  -0.9603     0.2626  -3.657 0.000255 ***
## x             2.0264     0.4230   4.791 1.66e-06 ***
## ---
## Signif. codes:  0 '***' 0.001 '**' 0.01 '*' 0.05 '.' 0.1 ' ' 1
##
## (Dispersion parameter for binomial family taken to be 1)
##
##     Null deviance: 345.28  on 249  degrees of freedom
## Residual deviance: 320.45  on 248  degrees of freedom
## AIC: 324.45
##
## Number of Fisher Scoring iterations: 4
##
## Subject id:  17              Estimate Std. Error   z value     Pr(>|z|)
## (Intercept) -0.9602651  0.2625946 -3.656834 2.553492e-04
## x            2.0264231  0.4229913  4.790697 1.662029e-06
## Subject id:  17 , Null model comparison (Chi)Analysis of Deviance Table
##
## Model: binomial, link: logit
##
## Response: y
##
## Terms added sequentially (first to last)
##
##
##      Df Deviance Resid. Df Resid. Dev  Pr(>Chi)
## NULL                   249     345.28
## x     1   24.826       248     320.45 6.275e-07 ***
## ---
## Signif. codes:  0 '***' 0.001 '**' 0.01 '*' 0.05 '.' 0.1 ' ' 1
## Subject id:  18 , Logistic Regression Model Summary
## Call:
## glm(formula = y ~ x, family = "binomial", data = sub1)
##
## Deviance Residuals:
##     Min       1Q   Median       3Q      Max
## -2.2072  -1.1478   0.4703   0.8654   1.1843
##
## Coefficients:
##             Estimate Std. Error z value Pr(>|z|)
## (Intercept) -0.07785    0.26751  -0.291    0.771
## x            2.57123    0.56742   4.531 5.86e-06 ***
## ---
```

```
## Signif. codes:  0 '***' 0.001 '**' 0.01 '*' 0.05 '.' 0.1 ' ' 1
##
## (Dispersion parameter for binomial family taken to be 1)
##
##     Null deviance: 284.42  on 249  degrees of freedom
## Residual deviance: 259.84  on 248  degrees of freedom
## AIC: 263.84
##
## Number of Fisher Scoring iterations: 4
##
## Subject id:  18                Estimate Std. Error    z value     Pr(>|z|)
## (Intercept) -0.07784533  0.2675068 -0.2910032 7.710489e-01
## x            2.57122706  0.5674164  4.5314641 5.857630e-06
## Subject id:  18 , Null model comparison (Chi)Analysis of Deviance Table
##
## Model: binomial, link: logit
##
## Response: y
##
## Terms added sequentially (first to last)
##
##
##      Df Deviance Resid. Df Resid. Dev  Pr(>Chi)
## NULL                   249     284.42
## x     1   24.575       248     259.84 7.146e-07 ***
## ---
## Signif. codes:  0 '***' 0.001 '**' 0.01 '*' 0.05 '.' 0.1 ' ' 1
```

```r
p = length(levels(data.sev$id))
Sev.collect <- list()
Sev.OR.collect <- list()
Sev.p.OR.collect <- list()
Sev.confint.collect <- list()

library(MuMIn)
library(DescTools)
for (i in 1:p){
  lev = levels(data.sev$id)[i]
  sub1 <- data.sev[data.sev$id==lev,]
  model = glm(y~x,family = "binomial",data = sub1)
  Sev.collect[[i]] = model
  Sev.OR.collect[[i]] <- exp(model$coefficients[2])
  Sev.p.OR.collect[[i]] <- summary(model)$coefficients[2,4]
  flush.console()
  Sev.confint.collect[[i]] <- confint.default(model)
  cat("Subject id: ", levels(data.sev$id)[i], ", Logistic Regression Model Summary")
  print(summary(model))
  cat("Subject id: ", levels(data.sev$id)[i])
  print(summary(model)$coefficients)
  cat("Subject id: ", levels(data.sev$id)[i], ", Null model comparison (Chi)")
  print(anova(model, test = 'Chi'))
}
```

```
## Subject id:  6 , Logistic Regression Model Summary
```

```
## Call:
## glm(formula = y ~ x, family = "binomial", data = sub1)
##
## Deviance Residuals:
##     Min       1Q   Median       3Q      Max
## -1.3809  -0.8373  -0.6427   1.0872   1.9090
##
## Coefficients:
##             Estimate Std. Error z value Pr(>|z|)
## (Intercept)  -1.8045     0.2736  -6.594 4.27e-11 ***
## x             2.5178     0.5448   4.621 3.81e-06 ***
## ---
## Signif. codes:  0 '***' 0.001 '**' 0.01 '*' 0.05 '.' 0.1 ' ' 1
##
## (Dispersion parameter for binomial family taken to be 1)
##
##     Null deviance: 310.35  on 249  degrees of freedom
## Residual deviance: 287.49  on 248  degrees of freedom
## AIC: 291.49
##
## Number of Fisher Scoring iterations: 4
##
## Subject id:  6             Estimate Std. Error   z value     Pr(>|z|)
## (Intercept) -1.804467  0.2736389 -6.594337 4.271593e-11
## x            2.517765  0.5448158  4.621314 3.813166e-06
## Subject id:  6 , Null model comparison (Chi)Analysis of Deviance Table
##
## Model: binomial, link: logit
##
## Response: y
##
## Terms added sequentially (first to last)
##
##
##      Df Deviance Resid. Df Resid. Dev Pr(>Chi)
## NULL                   249     310.35
## x     1   22.851       248     287.49 1.75e-06 ***
## ---
## Signif. codes:  0 '***' 0.001 '**' 0.01 '*' 0.05 '.' 0.1 ' ' 1
## Subject id:  7 , Logistic Regression Model Summary
## Call:
## glm(formula = y ~ x, family = "binomial", data = sub1)
##
## Deviance Residuals:
##     Min       1Q   Median       3Q      Max
## -0.9135  -0.7972  -0.5848  -0.4865   2.0997
##
## Coefficients:
##             Estimate Std. Error z value Pr(>|z|)
## (Intercept)  -0.6582     0.2468  -2.667  0.00766 **
## x            -1.6005     0.5840  -2.741  0.00613 **
## ---
## Signif. codes:  0 '***' 0.001 '**' 0.01 '*' 0.05 '.' 0.1 ' ' 1
##
```

```
## (Dispersion parameter for binomial family taken to be 1)
##
##     Null deviance: 265.96  on 249  degrees of freedom
## Residual deviance: 257.81  on 248  degrees of freedom
## AIC: 261.81
##
## Number of Fisher Scoring iterations: 4
##
## Subject id:  7              Estimate Std. Error   z value    Pr(>|z|)
## (Intercept) -0.6581659  0.2468164 -2.666622 0.007661777
## x           -1.6004804  0.5840030 -2.740534 0.006133935
## Subject id:  7 , Null model comparison (Chi)Analysis of Deviance Table
##
## Model: binomial, link: logit
##
## Response: y
##
## Terms added sequentially (first to last)
##
##
##      Df Deviance Resid. Df Resid. Dev Pr(>Chi)
## NULL                   249     265.96
## x     1   8.1556       248     257.81 0.004293 **
## ---
## Signif. codes:  0 '***' 0.001 '**' 0.01 '*' 0.05 '.' 0.1 ' ' 1
## Subject id:  9 , Logistic Regression Model Summary
## Call:
## glm(formula = y ~ x, family = "binomial", data = sub1)
##
## Deviance Residuals:
##     Min       1Q   Median       3Q      Max
## -1.2583  -0.9494  -0.8389   1.2702   1.6239
##
## Coefficients:
##             Estimate Std. Error z value Pr(>|z|)
## (Intercept)  -1.0072     0.2310  -4.359 1.31e-05 ***
## x             1.2951     0.4618   2.804  0.00505 **
## ---
## Signif. codes:  0 '***' 0.001 '**' 0.01 '*' 0.05 '.' 0.1 ' ' 1
##
## (Dispersion parameter for binomial family taken to be 1)
##
##     Null deviance: 332.03  on 249  degrees of freedom
## Residual deviance: 324.02  on 248  degrees of freedom
## AIC: 328.02
##
## Number of Fisher Scoring iterations: 4
##
## Subject id:  9             Estimate Std. Error   z value     Pr(>|z|)
## (Intercept) -1.007165  0.2310422 -4.359223 1.305248e-05
## x            1.295081  0.4618452  2.804145 5.045026e-03
## Subject id:  9 , Null model comparison (Chi)Analysis of Deviance Table
##
## Model: binomial, link: logit
```

```
##
## Response: y
##
## Terms added sequentially (first to last)
##
##
##      Df Deviance Resid. Df Resid. Dev Pr(>Chi)
## NULL                   249     332.03
## x     1   8.0122       248     324.02 0.004646 **
## ---
## Signif. codes:  0 '***' 0.001 '**' 0.01 '*' 0.05 '.' 0.1 ' ' 1
## Subject id:  14 , Logistic Regression Model Summary
## Call:
## glm(formula = y ~ x, family = "binomial", data = sub1)
##
## Deviance Residuals:
##     Min       1Q   Median       3Q      Max
## -1.6721  -0.9424  -0.7118   1.0170   1.8054
##
## Coefficients:
##             Estimate Std. Error z value Pr(>|z|)
## (Intercept)  -1.4172     0.2467  -5.745  9.2e-09 ***
## x             2.7970     0.5170   5.410  6.3e-08 ***
## ---
## Signif. codes:  0 '***' 0.001 '**' 0.01 '*' 0.05 '.' 0.1 ' ' 1
##
## (Dispersion parameter for binomial family taken to be 1)
##
##     Null deviance: 340.15  on 249  degrees of freedom
## Residual deviance: 307.39  on 248  degrees of freedom
## AIC: 311.39
##
## Number of Fisher Scoring iterations: 4
##
## Subject id:  14             Estimate Std. Error   z value     Pr(>|z|)
## (Intercept) -1.417239  0.2466960 -5.744879 9.198694e-09
## x            2.797020  0.5170065  5.410029 6.301451e-08
## Subject id:  14 , Null model comparison (Chi)Analysis of Deviance Table
##
## Model: binomial, link: logit
##
## Response: y
##
## Terms added sequentially (first to last)
##
##
##      Df Deviance Resid. Df Resid. Dev  Pr(>Chi)
## NULL                   249     340.15
## x     1   32.759       248     307.39 1.043e-08 ***
## ---
## Signif. codes:  0 '***' 0.001 '**' 0.01 '*' 0.05 '.' 0.1 ' ' 1
## Subject id:  15 , Logistic Regression Model Summary
## Call:
## glm(formula = y ~ x, family = "binomial", data = sub1)
```

```
##
## Deviance Residuals:
##     Min       1Q   Median       3Q      Max
## -0.9192  -0.8003  -0.7345   1.4723   1.7432
##
## Coefficients:
##             Estimate Std. Error z value Pr(>|z|)
## (Intercept)  -1.2828     0.2501   -5.13  2.9e-07 ***
## x             0.6930     0.4950    1.40    0.162
## ---
## Signif. codes:  0 '***' 0.001 '**' 0.01 '*' 0.05 '.' 0.1 ' ' 1
##
## (Dispersion parameter for binomial family taken to be 1)
##
##     Null deviance: 290.63  on 249  degrees of freedom
## Residual deviance: 288.68  on 248  degrees of freedom
## AIC: 292.68
##
## Number of Fisher Scoring iterations: 4
##
## Subject id:  15              Estimate Std. Error   z value     Pr(>|z|)
## (Intercept) -1.2828095  0.2500690 -5.129821 2.900176e-07
## x            0.6930361  0.4950186  1.400020 1.615072e-01
## Subject id:  15 , Null model comparison (Chi)Analysis of Deviance Table
##
## Model: binomial, link: logit
##
## Response: y
##
## Terms added sequentially (first to last)
##
##
##      Df Deviance Resid. Df Resid. Dev Pr(>Chi)
## NULL                   249     290.63
## x     1    1.954       248     288.68   0.1622
## Subject id:  19 , Logistic Regression Model Summary
## Call:
## glm(formula = y ~ x, family = "binomial", data = sub1)
##
## Deviance Residuals:
##     Min       1Q   Median       3Q      Max
## -1.5170  -0.8989  -0.6889   1.0845   1.9102
##
## Coefficients:
##             Estimate Std. Error z value Pr(>|z|)
## (Intercept)  -1.6486     0.2637  -6.251 4.07e-10 ***
## x             2.7487     0.5593   4.915 8.89e-07 ***
## ---
## Signif. codes:  0 '***' 0.001 '**' 0.01 '*' 0.05 '.' 0.1 ' ' 1
##
## (Dispersion parameter for binomial family taken to be 1)
##
##     Null deviance: 325.54  on 249  degrees of freedom
## Residual deviance: 299.25  on 248  degrees of freedom
```

```
## AIC: 303.25
##
## Number of Fisher Scoring iterations: 4
##
## Subject id:  19             Estimate Std. Error   z value     Pr(>|z|)
## (Intercept) -1.648621  0.2637171 -6.251475 4.065936e-10
## x            2.748712  0.5592731  4.914794 8.887594e-07
## Subject id:  19 , Null model comparison (Chi)Analysis of Deviance Table
##
## Model: binomial, link: logit
##
## Response: y
##
## Terms added sequentially (first to last)
##
##
##      Df Deviance Resid. Df Resid. Dev  Pr(>Chi)
## NULL                   249     325.54
## x     1   26.291       248     299.25 2.937e-07 ***
## ---
## Signif. codes:  0 '***' 0.001 '**' 0.01 '*' 0.05 '.' 0.1 ' ' 1
## Subject id:  20 , Logistic Regression Model Summary
## Call:
## glm(formula = y ~ x, family = "binomial", data = sub1)
##
## Deviance Residuals:
##     Min       1Q   Median       3Q      Max
## -1.5126  -0.9055  -0.7522   1.1311   1.7865
##
## Coefficients:
##             Estimate Std. Error z value Pr(>|z|)
## (Intercept)  -1.3716     0.2490  -5.509 3.61e-08 ***
## x             2.3612     0.5235   4.510 6.47e-06 ***
## ---
## Signif. codes:  0 '***' 0.001 '**' 0.01 '*' 0.05 '.' 0.1 ' ' 1
##
## (Dispersion parameter for binomial family taken to be 1)
##
##     Null deviance: 333.92  on 249  degrees of freedom
## Residual deviance: 312.11  on 248  degrees of freedom
## AIC: 316.11
##
## Number of Fisher Scoring iterations: 4
##
## Subject id:  20             Estimate Std. Error  z value     Pr(>|z|)
## (Intercept) -1.371607  0.2489875 -5.50874 3.614122e-08
## x            2.361163  0.5234931  4.51040 6.470550e-06
## Subject id:  20 , Null model comparison (Chi)Analysis of Deviance Table
##
## Model: binomial, link: logit
##
## Response: y
##
## Terms added sequentially (first to last)
```

```
##
##
##      Df Deviance Resid. Df Resid. Dev  Pr(>Chi)
## NULL                   249     333.92
## x     1   21.815       248     312.11 3.002e-06 ***
## ---
## Signif. codes:  0 '***' 0.001 '**' 0.01 '*' 0.05 '.' 0.1 ' ' 1
```

Experiment 2

```
p = length(levels(data.RLV$id))
RLV.collect <- list()
RLV.Slope.collect <-list()
RLV.OR.collect <- list()
RLV.p.OR.collect <- list()
RLV.confint.collect <- list()

library(MuMIn)
library(DescTools)
for (i in 1:p){
  lev = levels(data.RLV$id)[i]
  sub1 <- data.RLV[data.RLV$id==lev,]
  model = glm(y~x,family = "binomial",data = sub1)
  RLV.collect[[i]] = model
  RLV.Slope.collect[[i]] <- model$coefficients[2]
  RLV.OR.collect[[i]] <- exp(model$coefficients[2])
  RLV.p.OR.collect[[i]] <- summary(model)$coefficients[2,4]
  RLV.confint.collect[[i]] <- confint.default(model)
  cat("Subject id: ", levels(data.RLV$id)[i], ", Logistic Regression Model Summary")
  print(summary(model))
  cat("Subject id: ", levels(data.RLV$id)[i])
  print(summary(model)$coefficients)
  cat("Subject id: ", levels(data.RLV$id)[i], ", Null model comparison (Chi)")
  print(anova(model, test = 'Chi'))
}
```

```
## Subject id:  2 , Logistic Regression Model Summary
## Call:
## glm(formula = y ~ x, family = "binomial", data = sub1)
##
## Deviance Residuals:
##     Min       1Q   Median       3Q      Max
## -3.6461   0.0502   0.0508   0.3231   0.6780
##
## Coefficients:
##             Estimate Std. Error z value Pr(>|z|)
## (Intercept)   0.2497     0.8355   0.299  0.76505
## x             6.4541     2.3176   2.785  0.00536 **
## ---
## Signif. codes:  0 '***' 0.001 '**' 0.01 '*' 0.05 '.' 0.1 ' ' 1
##
## (Dispersion parameter for binomial family taken to be 1)
```

```
##
##     Null deviance: 70.814  on 249  degrees of freedom
## Residual deviance: 52.825  on 248  degrees of freedom
## AIC: 56.825
##
## Number of Fisher Scoring iterations: 8
##
## Subject id:  2             Estimate Std. Error   z value    Pr(>|z|)
## (Intercept) 0.2497061  0.8355283 0.2988602 0.765046735
## x           6.4540579  2.3175790 2.7848275 0.005355621
## Subject id:  2 , Null model comparison (Chi)Analysis of Deviance Table
##
## Model: binomial, link: logit
##
## Response: y
##
## Terms added sequentially (first to last)
##
##
##      Df Deviance Resid. Df Resid. Dev  Pr(>Chi)
## NULL                   249     70.814
## x     1   17.989       248     52.825 2.222e-05 ***
## ---
## Signif. codes:  0 '***' 0.001 '**' 0.01 '*' 0.05 '.' 0.1 ' ' 1
## Subject id:  3 , Logistic Regression Model Summary
## Call:
## glm(formula = y ~ x, family = "binomial", data = sub1)
##
## Deviance Residuals:
##     Min       1Q   Median       3Q      Max
## -2.0683   0.4940   0.5424   0.8033   0.9482
##
## Coefficients:
##             Estimate Std. Error z value Pr(>|z|)
## (Intercept)   0.5035     0.2678    1.88  0.06012 .
## x             1.5714     0.5134    3.06  0.00221 **
## ---
## Signif. codes:  0 '***' 0.001 '**' 0.01 '*' 0.05 '.' 0.1 ' ' 1
##
## (Dispersion parameter for binomial family taken to be 1)
##
##     Null deviance: 265.96  on 249  degrees of freedom
## Residual deviance: 255.75  on 248  degrees of freedom
## AIC: 259.75
##
## Number of Fisher Scoring iterations: 4
##
## Subject id:  3             Estimate Std. Error  z value    Pr(>|z|)
## (Intercept) 0.5034699   0.267816 1.879910 0.060120378
## x           1.5713808   0.513449 3.060442 0.002210108
## Subject id:  3 , Null model comparison (Chi)Analysis of Deviance Table
##
## Model: binomial, link: logit
##
```

```
## Response: y
##
## Terms added sequentially (first to last)
##
##
##      Df Deviance Resid. Df Resid. Dev Pr(>Chi)
## NULL                   249     265.96
## x     1    10.21       248     255.75 0.001397 **
## ---
## Signif. codes:  0 '***' 0.001 '**' 0.01 '*' 0.05 '.' 0.1 ' ' 1
## Subject id:  4 , Logistic Regression Model Summary
## Call:
## glm(formula = y ~ x, family = "binomial", data = sub1)
##
## Deviance Residuals:
##     Min       1Q   Median       3Q      Max
## -1.8273  -0.8351  -0.6713   0.6691   1.7893
##
## Coefficients:
##             Estimate Std. Error z value Pr(>|z|)
## (Intercept)  -1.3754     0.1990  -6.911 4.81e-12 ***
## x             3.2339     0.4766   6.785 1.16e-11 ***
## ---
## Signif. codes:  0 '***' 0.001 '**' 0.01 '*' 0.05 '.' 0.1 ' ' 1
##
## (Dispersion parameter for binomial family taken to be 1)
##
##     Null deviance: 337.30  on 249  degrees of freedom
## Residual deviance: 276.59  on 248  degrees of freedom
## AIC: 280.59
##
## Number of Fisher Scoring iterations: 4
##
## Subject id:  4             Estimate Std. Error   z value     Pr(>|z|)
## (Intercept) -1.375394  0.1990115 -6.911126 4.808217e-12
## x            3.233945  0.4766322  6.784992 1.160932e-11
## Subject id:  4 , Null model comparison (Chi)Analysis of Deviance Table
##
## Model: binomial, link: logit
##
## Response: y
##
## Terms added sequentially (first to last)
##
##
##      Df Deviance Resid. Df Resid. Dev  Pr(>Chi)
## NULL                   249     337.30
## x     1   60.709       248     276.59 6.615e-15 ***
## ---
## Signif. codes:  0 '***' 0.001 '**' 0.01 '*' 0.05 '.' 0.1 ' ' 1
## Subject id:  5 , Logistic Regression Model Summary
## Call:
## glm(formula = y ~ x, family = "binomial", data = sub1)
##
```

```
## Deviance Residuals:
##     Min       1Q   Median       3Q      Max
## -2.8466   0.1748   0.1999   0.5623   1.5099
##
## Coefficients:
##             Estimate Std. Error z value Pr(>|z|)
## (Intercept)  -1.3985     0.3424  -4.085 4.41e-05 ***
## x             5.6006     0.8135   6.884 5.81e-12 ***
## ---
## Signif. codes:  0 '***' 0.001 '**' 0.01 '*' 0.05 '.' 0.1 ' ' 1
##
## (Dispersion parameter for binomial family taken to be 1)
##
##     Null deviance: 265.96  on 249  degrees of freedom
## Residual deviance: 178.18  on 248  degrees of freedom
## AIC: 182.18
##
## Number of Fisher Scoring iterations: 6
##
## Subject id:  5             Estimate Std. Error   z value     Pr(>|z|)
## (Intercept) -1.398519  0.3423546 -4.085000 4.407673e-05
## x            5.600629  0.8135421  6.884252 5.809187e-12
## Subject id:  5 , Null model comparison (Chi)Analysis of Deviance Table
##
## Model: binomial, link: logit
##
## Response: y
##
## Terms added sequentially (first to last)
##
##
##      Df Deviance Resid. Df Resid. Dev  Pr(>Chi)
## NULL                   249     265.96
## x     1    87.78       248     178.18 < 2.2e-16 ***
## ---
## Signif. codes:  0 '***' 0.001 '**' 0.01 '*' 0.05 '.' 0.1 ' ' 1
## Subject id:  6 , Logistic Regression Model Summary
## Call:
## glm(formula = y ~ x, family = "binomial", data = sub1)
##
## Deviance Residuals:
##     Min       1Q   Median       3Q      Max
## -1.7858  -0.9540   0.2510   0.9519   1.6365
##
## Coefficients:
##             Estimate Std. Error z value Pr(>|z|)
## (Intercept)  -1.0351     0.2296  -4.509 6.51e-06 ***
## x             5.5619     0.8612   6.458 1.06e-10 ***
## ---
## Signif. codes:  0 '***' 0.001 '**' 0.01 '*' 0.05 '.' 0.1 ' ' 1
##
## (Dispersion parameter for binomial family taken to be 1)
##
##     Null deviance: 338.79  on 249  degrees of freedom
```

```
## Residual deviance: 267.44  on 248  degrees of freedom
## AIC: 271.44
##
## Number of Fisher Scoring iterations: 5
##
## Subject id:  6             Estimate Std. Error   z value     Pr(>|z|)
## (Intercept) -1.035114  0.2295579 -4.509164 6.508361e-06
## x            5.561864  0.8612027  6.458252 1.059190e-10
## Subject id:  6 , Null model comparison (Chi)Analysis of Deviance Table
##
## Model: binomial, link: logit
##
## Response: y
##
## Terms added sequentially (first to last)
##
##
##      Df Deviance Resid. Df Resid. Dev  Pr(>Chi)
## NULL                   249     338.79
## x     1   71.346       248     267.44 < 2.2e-16 ***
## ---
## Signif. codes:  0 '***' 0.001 '**' 0.01 '*' 0.05 '.' 0.1 ' ' 1
## Subject id:  7 , Logistic Regression Model Summary
## Call:
## glm(formula = y ~ x, family = "binomial", data = sub1)
##
## Deviance Residuals:
##     Min       1Q   Median       3Q      Max
## -2.2676  -1.0139   0.4484   0.8420   1.6850
##
## Coefficients:
##             Estimate Std. Error z value Pr(>|z|)
## (Intercept)  -1.1505     0.2709  -4.247 2.17e-05 ***
## x             3.7976     0.5672   6.696 2.14e-11 ***
## ---
## Signif. codes:  0 '***' 0.001 '**' 0.01 '*' 0.05 '.' 0.1 ' ' 1
##
## (Dispersion parameter for binomial family taken to be 1)
##
##     Null deviance: 326.71  on 249  degrees of freedom
## Residual deviance: 266.45  on 248  degrees of freedom
## AIC: 270.45
##
## Number of Fisher Scoring iterations: 4
##
## Subject id:  7             Estimate Std. Error   z value     Pr(>|z|)
## (Intercept) -1.150492  0.2708911 -4.247065 2.165887e-05
## x            3.797580  0.5671508  6.695891 2.143618e-11
## Subject id:  7 , Null model comparison (Chi)Analysis of Deviance Table
##
## Model: binomial, link: logit
##
## Response: y
##
```

```
## Terms added sequentially (first to last)
##
##
##      Df Deviance Resid. Df Resid. Dev  Pr(>Chi)
## NULL                   249     326.71
## x     1   60.264       248     266.45 8.296e-15 ***
## ---
## Signif. codes:  0 '***' 0.001 '**' 0.01 '*' 0.05 '.' 0.1 ' ' 1
## Subject id:  8 , Logistic Regression Model Summary
## Call:
## glm(formula = y ~ x, family = "binomial", data = sub1)
##
## Deviance Residuals:
##     Min       1Q   Median       3Q      Max
## -1.8034  -1.0392  -0.9899   1.1973   1.3773
##
## Coefficients:
##             Estimate Std. Error z value Pr(>|z|)
## (Intercept)  -0.4586     0.1631  -2.812  0.00492 **
## x             2.2725     0.5726   3.969 7.22e-05 ***
## ---
## Signif. codes:  0 '***' 0.001 '**' 0.01 '*' 0.05 '.' 0.1 ' ' 1
##
## (Dispersion parameter for binomial family taken to be 1)
##
##     Null deviance: 346.51  on 249  degrees of freedom
## Residual deviance: 327.50  on 248  degrees of freedom
## AIC: 331.5
##
## Number of Fisher Scoring iterations: 4
##
## Subject id:  8              Estimate Std. Error   z value     Pr(>|z|)
## (Intercept) -0.4586127  0.1630671 -2.812417 4.917077e-03
## x            2.2725028  0.5725789  3.968891 7.220803e-05
## Subject id:  8 , Null model comparison (Chi)Analysis of Deviance Table
##
## Model: binomial, link: logit
##
## Response: y
##
## Terms added sequentially (first to last)
##
##
##      Df Deviance Resid. Df Resid. Dev  Pr(>Chi)
## NULL                   249     346.51
## x     1   19.014       248     327.50 1.298e-05 ***
## ---
## Signif. codes:  0 '***' 0.001 '**' 0.01 '*' 0.05 '.' 0.1 ' ' 1
## Subject id:  9 , Logistic Regression Model Summary
## Call:
## glm(formula = y ~ x, family = "binomial", data = sub1)
##
## Deviance Residuals:
##     Min       1Q   Median       3Q      Max
```

```
## -1.7611  -0.9198  -0.7042   0.9408   1.8336
##
## Coefficients:
##             Estimate Std. Error z value Pr(>|z|)
## (Intercept)  -1.4751     0.2542  -5.804 6.48e-09 ***
## x             3.0430     0.5029   6.051 1.44e-09 ***
## ---
## Signif. codes:  0 '***' 0.001 '**' 0.01 '*' 0.05 '.' 0.1 ' ' 1
##
## (Dispersion parameter for binomial family taken to be 1)
##
##     Null deviance: 344.27  on 249  degrees of freedom
## Residual deviance: 302.38  on 248  degrees of freedom
## AIC: 306.38
##
## Number of Fisher Scoring iterations: 4
##
## Subject id:  9             Estimate Std. Error   z value     Pr(>|z|)
## (Intercept) -1.475100  0.2541594 -5.803838 6.481397e-09
## x            3.043047  0.5029242  6.050707 1.442116e-09
## Subject id:  9 , Null model comparison (Chi)Analysis of Deviance Table
##
## Model: binomial, link: logit
##
## Response: y
##
## Terms added sequentially (first to last)
##
##
##      Df Deviance Resid. Df Resid. Dev  Pr(>Chi)
## NULL                   249     344.27
## x     1   41.891       248     302.38 9.651e-11 ***
## ---
## Signif. codes:  0 '***' 0.001 '**' 0.01 '*' 0.05 '.' 0.1 ' ' 1
## Subject id:  10 , Logistic Regression Model Summary
## Call:
## glm(formula = y ~ x, family = "binomial", data = sub1)
##
## Deviance Residuals:
##     Min       1Q   Median       3Q      Max
## -1.4027  -0.9030  -0.7093   1.0489   1.8700
##
## Coefficients:
##             Estimate Std. Error z value Pr(>|z|)
## (Intercept)  -1.5852     0.2624  -6.041 1.53e-09 ***
## x             2.5372     0.5568   4.557 5.19e-06 ***
## ---
## Signif. codes:  0 '***' 0.001 '**' 0.01 '*' 0.05 '.' 0.1 ' ' 1
##
## (Dispersion parameter for binomial family taken to be 1)
##
##     Null deviance: 324.34  on 249  degrees of freedom
## Residual deviance: 302.10  on 248  degrees of freedom
## AIC: 306.1
```

```
##
## Number of Fisher Scoring iterations: 4
##
## Subject id:  10             Estimate Std. Error   z value     Pr(>|z|)
## (Intercept) -1.585231  0.2624128 -6.040980 1.531810e-09
## x            2.537239  0.5567610  4.557142 5.185436e-06
## Subject id:  10 , Null model comparison (Chi)Analysis of Deviance Table
##
## Model: binomial, link: logit
##
## Response: y
##
## Terms added sequentially (first to last)
##
##
##      Df Deviance Resid. Df Resid. Dev  Pr(>Chi)
## NULL                   249     324.34
## x     1   22.238       248     302.10 2.409e-06 ***
## ---
## Signif. codes:  0 '***' 0.001 '**' 0.01 '*' 0.05 '.' 0.1 ' ' 1
## Subject id:  11 , Logistic Regression Model Summary
## Call:
## glm(formula = y ~ x, family = "binomial", data = sub1)
##
## Deviance Residuals:
##      Min        1Q    Median        3Q       Max
## -2.35688   0.01246   0.01309   0.30550   1.30155
##
## Coefficients:
##             Estimate Std. Error z value Pr(>|z|)
## (Intercept)  -1.4565     0.7738  -1.882 0.059815 .
## x            11.0309     2.9492   3.740 0.000184 ***
## ---
## Signif. codes:  0 '***' 0.001 '**' 0.01 '*' 0.05 '.' 0.1 ' ' 1
##
## (Dispersion parameter for binomial family taken to be 1)
##
##     Null deviance: 124.217  on 249  degrees of freedom
## Residual deviance:  72.188  on 248  degrees of freedom
## AIC: 76.188
##
## Number of Fisher Scoring iterations: 8
##
## Subject id:  11            Estimate Std. Error   z value     Pr(>|z|)
## (Intercept) -1.45646  0.7738247 -1.882158 0.0598145964
## x           11.03090  2.9491512  3.740364 0.0001837537
## Subject id:  11 , Null model comparison (Chi)Analysis of Deviance Table
##
## Model: binomial, link: logit
##
## Response: y
##
## Terms added sequentially (first to last)
##
```

```
##
##      Df Deviance Resid. Df Resid. Dev  Pr(>Chi)
## NULL                    249    124.217
## x     1   52.029       248     72.188 5.469e-13 ***
## ---
## Signif. codes:  0 '***' 0.001 '**' 0.01 '*' 0.05 '.' 0.1 ' ' 1
```

Correlation between slope values and VA/CS of participants

```
RLV.slopes <- data.frame(data.frame(matrix(unlist(RLV.Slope.collect),nrow=length(RLV.Slope.collect), by
RLV.SLVC <- cbind(data.frame(RLV.slopes),RLV.VA, RLV.CS)
colnames(RLV.SLVC) <- c("slopes","VA","CS")
model.RLV.VASL <- lm(slopes~VA, data = RLV.SLVC)
summary(model.RLV.VASL)
```

```
##
## Call:
## lm(formula = slopes ~ VA, data = RLV.SLVC)
##
## Residuals:
##     Min      1Q  Median      3Q     Max
## -3.0014 -1.2549 -0.0902  0.3237  4.8334
##
## Coefficients:
##             Estimate Std. Error t value Pr(>|t|)
## (Intercept)   12.571      3.994   3.148   0.0136 *
## VA            -6.249      3.040  -2.056   0.0738 .
## ---
## Signif. codes:  0 '***' 0.001 '**' 0.01 '*' 0.05 '.' 0.1 ' ' 1
##
## Residual standard error: 2.4 on 8 degrees of freedom
## Multiple R-squared:  0.3457, Adjusted R-squared:  0.2639
## F-statistic: 4.226 on 1 and 8 DF,  p-value: 0.07384
```

```
model.RLV.CSSL <- lm(slopes~CS, data = RLV.SLVC)
summary(model.RLV.CSSL)
```

```
##
## Call:
## lm(formula = slopes ~ CS, data = RLV.SLVC)
##
## Residuals:
##     Min      1Q  Median      3Q     Max
## -2.2504 -1.1169 -0.4626  0.5117  3.2924
##
## Coefficients:
##             Estimate Std. Error t value Pr(>|t|)
## (Intercept)    1.348      1.174   1.148    0.284
## CS             4.123      1.297   3.178    0.013 *
## ---
## Signif. codes:  0 '***' 0.001 '**' 0.01 '*' 0.05 '.' 0.1 ' ' 1
##
```

```
## Residual standard error: 1.972 on 8 degrees of freedom
## Multiple R-squared:  0.5581, Adjusted R-squared:  0.5028
## F-statistic:  10.1 on 1 and 8 DF,  p-value: 0.01303
```

###RLV individual, with Central ROI

```
p = length(levels(data.RLVC$id))
RLVC.collect <- list()
RLVC.Slope.collect <- list()
RLVC.OR.collect <- list()
RLVC.p.OR.collect <- list()
RLVC.confint.collect <- list()

library(MuMIn)
library(DescTools)
for (i in 1:p){
  lev = levels(data.RLVC$id)[i]
  sub1 <- data.RLVC[data.RLVC$id==lev,]
  model = glm(y~x,family = "binomial",data = sub1)
  RLVC.collect[[i]] = model
  RLVC.Slope.collect[[i]] <- model$coefficients[2]
  RLVC.OR.collect[[i]] <- exp(model$coefficients[2])
  RLVC.p.OR.collect[[i]] <- summary(model)$coefficients[2,4]
  RLVC.confint.collect[[i]] <- confint.default(model)
  cat("Subject id: ", levels(data.RLVC$id)[i], ", Logistic Regression Model Summary")
  print(summary(model))
  cat("Subject id: ", levels(data.RLVC$id)[i], ", Coefficients, SE, and CI")
  print(summary(model)$coefficients)
  cat("Subject id: ", levels(data.RLVC$id)[i], ", Null model comparison (Chi)")
  print(anova(model, test = 'Chi'))
}
```

```
## Subject id:  2 , Logistic Regression Model Summary
## Call:
## glm(formula = y ~ x, family = "binomial", data = sub1)
##
## Deviance Residuals:
##     Min       1Q   Median       3Q      Max
## -3.4105   0.0761   0.0771   0.4079   0.5180
##
## Coefficients:
##             Estimate Std. Error z value Pr(>|z|)
## (Intercept)   1.6513     0.5168   3.195   0.0014 **
## x             4.1992     1.8005   2.332   0.0197 *
## ---
## Signif. codes:  0 '***' 0.001 '**' 0.01 '*' 0.05 '.' 0.1 ' ' 1
##
## (Dispersion parameter for binomial family taken to be 1)
##
##     Null deviance: 68.732  on 219  degrees of freedom
## Residual deviance: 56.234  on 218  degrees of freedom
##   (30 observations deleted due to missingness)
## AIC: 60.234
```

```
##
## Number of Fisher Scoring iterations: 8
##
## Subject id:  2 , Coefficients, SE, and CI            Estimate Std. Error  z value   Pr(>|z|)
## (Intercept) 1.651274  0.5168428 3.194925 0.00139867
## x           4.199168  1.8005368 2.332176 0.01969145
## Subject id:  2 , Null model comparison (Chi)Analysis of Deviance Table
##
## Model: binomial, link: logit
##
## Response: y
##
## Terms added sequentially (first to last)
##
##
##      Df Deviance Resid. Df Resid. Dev  Pr(>Chi)
## NULL                   219     68.732
## x     1   12.498       218     56.234 0.0004073 ***
## ---
## Signif. codes:  0 '***' 0.001 '**' 0.01 '*' 0.05 '.' 0.1 ' ' 1
## Subject id:  3 , Logistic Regression Model Summary
## Call:
## glm(formula = y ~ x, family = "binomial", data = sub1)
##
## Deviance Residuals:
##     Min       1Q   Median       3Q      Max
## -2.4306   0.3211   0.4026   0.7954   0.8866
##
## Coefficients:
##             Estimate Std. Error z value Pr(>|z|)
## (Intercept)   0.6946     0.2261   3.072  0.00213 **
## x             2.2669     0.6154   3.683  0.00023 ***
## ---
## Signif. codes:  0 '***' 0.001 '**' 0.01 '*' 0.05 '.' 0.1 ' ' 1
##
## (Dispersion parameter for binomial family taken to be 1)
##
##     Null deviance: 217.38  on 219  degrees of freedom
## Residual deviance: 198.75  on 218  degrees of freedom
##   (30 observations deleted due to missingness)
## AIC: 202.75
##
## Number of Fisher Scoring iterations: 5
##
## Subject id:  3 , Coefficients, SE, and CI             Estimate Std. Error  z value     Pr(>|z|)
## (Intercept) 0.6946471  0.2261367 3.071801 0.0021277138
## x           2.2669134  0.6154319 3.683451 0.0002300972
## Subject id:  3 , Null model comparison (Chi)Analysis of Deviance Table
##
## Model: binomial, link: logit
##
## Response: y
##
## Terms added sequentially (first to last)
```

```
##
##
##      Df Deviance Resid. Df Resid. Dev  Pr(>Chi)
## NULL                   219     217.38
## x     1    18.63       218     198.75 1.587e-05 ***
## ---
## Signif. codes:  0 '***' 0.001 '**' 0.01 '*' 0.05 '.' 0.1 ' ' 1
## Subject id:  4 , Logistic Regression Model Summary
## Call:
## glm(formula = y ~ x, family = "binomial", data = sub1)
##
## Deviance Residuals:
##     Min       1Q   Median       3Q      Max
## -2.3051  -0.7335  -0.6985   0.4013   1.7495
##
## Coefficients:
##             Estimate Std. Error z value Pr(>|z|)
## (Intercept)  -1.2864     0.1902  -6.765 1.33e-11 ***
## x             4.1131     0.6451   6.376 1.82e-10 ***
## ---
## Signif. codes:  0 '***' 0.001 '**' 0.01 '*' 0.05 '.' 0.1 ' ' 1
##
## (Dispersion parameter for binomial family taken to be 1)
##
##     Null deviance: 302.61  on 223  degrees of freedom
## Residual deviance: 225.32  on 222  degrees of freedom
##   (26 observations deleted due to missingness)
## AIC: 229.32
##
## Number of Fisher Scoring iterations: 5
##
## Subject id:  4 , Coefficients, SE, and CI             Estimate Std. Error   z value     Pr(>|z|)
## (Intercept) -1.286406  0.1901576 -6.764947 1.333580e-11
## x            4.113084  0.6451199  6.375688 1.821433e-10
## Subject id:  4 , Null model comparison (Chi)Analysis of Deviance Table
##
## Model: binomial, link: logit
##
## Response: y
##
## Terms added sequentially (first to last)
##
##
##      Df Deviance Resid. Df Resid. Dev  Pr(>Chi)
## NULL                   223     302.61
## x     1   77.284       222     225.32 < 2.2e-16 ***
## ---
## Signif. codes:  0 '***' 0.001 '**' 0.01 '*' 0.05 '.' 0.1 ' ' 1
## Subject id:  5 , Logistic Regression Model Summary
## Call:
## glm(formula = y ~ x, family = "binomial", data = sub1)
##
## Deviance Residuals:
##     Min       1Q   Median       3Q      Max
```

```
## -3.6420   0.0465   0.0577   0.3617   1.5204
##
## Coefficients:
##             Estimate Std. Error z value Pr(>|z|)
## (Intercept)  -0.7778     0.2745  -2.834   0.0046 **
## x             7.6143     1.6559   4.598 4.26e-06 ***
## ---
## Signif. codes:  0 '***' 0.001 '**' 0.01 '*' 0.05 '.' 0.1 ' ' 1
##
## (Dispersion parameter for binomial family taken to be 1)
##
##     Null deviance: 242.94  on 219  degrees of freedom
## Residual deviance: 142.84  on 218  degrees of freedom
##   (30 observations deleted due to missingness)
## AIC: 146.84
##
## Number of Fisher Scoring iterations: 7
##
## Subject id:  5 , Coefficients, SE, and CI              Estimate Std. Error   z value     Pr(>|z|)
## (Intercept) -0.7778212  0.2744744 -2.833858 4.598983e-03
## x            7.6142870  1.6558944  4.598292 4.259677e-06
## Subject id:  5 , Null model comparison (Chi)Analysis of Deviance Table
##
## Model: binomial, link: logit
##
## Response: y
##
## Terms added sequentially (first to last)
##
##
##      Df Deviance Resid. Df Resid. Dev  Pr(>Chi)
## NULL                   219     242.94
## x     1    100.1       218     142.84 < 2.2e-16 ***
## ---
## Signif. codes:  0 '***' 0.001 '**' 0.01 '*' 0.05 '.' 0.1 ' ' 1
## Subject id:  6 , Logistic Regression Model Summary
## Call:
## glm(formula = y ~ x, family = "binomial", data = sub1)
##
## Deviance Residuals:
##     Min       1Q   Median       3Q      Max
## -2.1420  -0.9421   0.1401   0.9520   1.6337
##
## Coefficients:
##             Estimate Std. Error z value Pr(>|z|)
## (Intercept)  -1.0289     0.2154  -4.776 1.79e-06 ***
## x             6.0349     1.0520   5.736 9.67e-09 ***
## ---
## Signif. codes:  0 '***' 0.001 '**' 0.01 '*' 0.05 '.' 0.1 ' ' 1
##
## (Dispersion parameter for binomial family taken to be 1)
##
##     Null deviance: 304.09  on 219  degrees of freedom
## Residual deviance: 234.49  on 218  degrees of freedom
```

```
##   (30 observations deleted due to missingness)
## AIC: 238.49
##
## Number of Fisher Scoring iterations: 5
##
## Subject id:  6 , Coefficients, SE, and CI             Estimate Std. Error   z value     Pr(>|z|)
## (Intercept) -1.028861  0.2154194 -4.776082 1.787438e-06
## x            6.034906  1.0520225  5.736480 9.666456e-09
## Subject id:  6 , Null model comparison (Chi)Analysis of Deviance Table
##
## Model: binomial, link: logit
##
## Response: y
##
## Terms added sequentially (first to last)
##
##
##      Df Deviance Resid. Df Resid. Dev  Pr(>Chi)
## NULL                   219     304.09
## x     1   69.602       218     234.49 < 2.2e-16 ***
## ---
## Signif. codes:  0 '***' 0.001 '**' 0.01 '*' 0.05 '.' 0.1 ' ' 1
## Subject id:  7 , Logistic Regression Model Summary
## Call:
## glm(formula = y ~ x, family = "binomial", data = sub1)
##
## Deviance Residuals:
##     Min       1Q   Median       3Q      Max
## -2.4831  -0.9614   0.3358   0.8075   1.5123
##
## Coefficients:
##             Estimate Std. Error z value Pr(>|z|)
## (Intercept)  -0.7598     0.2199  -3.456 0.000548 ***
## x             3.9295     0.6105   6.437 1.22e-10 ***
## ---
## Signif. codes:  0 '***' 0.001 '**' 0.01 '*' 0.05 '.' 0.1 ' ' 1
##
## (Dispersion parameter for binomial family taken to be 1)
##
##     Null deviance: 288.41  on 219  degrees of freedom
## Residual deviance: 219.70  on 218  degrees of freedom
##   (30 observations deleted due to missingness)
## AIC: 223.7
##
## Number of Fisher Scoring iterations: 5
##
## Subject id:  7 , Coefficients, SE, and CI             Estimate Std. Error   z value     Pr(>|z|)
## (Intercept) -0.759817  0.2198526 -3.456029 5.481956e-04
## x            3.929512  0.6104693  6.436872 1.219608e-10
## Subject id:  7 , Null model comparison (Chi)Analysis of Deviance Table
##
## Model: binomial, link: logit
##
## Response: y
```

```
##
## Terms added sequentially (first to last)
##
##
##      Df Deviance Resid. Df Resid. Dev  Pr(>Chi)
## NULL                   219     288.41
## x     1   68.713       218     219.70 < 2.2e-16 ***
## ---
## Signif. codes:  0 '***' 0.001 '**' 0.01 '*' 0.05 '.' 0.1 ' ' 1
## Subject id:  8 , Logistic Regression Model Summary
## Call:
## glm(formula = y ~ x, family = "binomial", data = sub1)
##
## Deviance Residuals:
##     Min       1Q   Median       3Q      Max
## -2.3814  -1.0395   0.3373   1.2019   1.3218
##
## Coefficients:
##             Estimate Std. Error z value Pr(>|z|)
## (Intercept)  -0.3333     0.1544  -2.159 0.030827 *
## x             3.3351     0.9045   3.687 0.000227 ***
## ---
## Signif. codes:  0 '***' 0.001 '**' 0.01 '*' 0.05 '.' 0.1 ' ' 1
##
## (Dispersion parameter for binomial family taken to be 1)
##
##     Null deviance: 321.55  on 231  degrees of freedom
## Residual deviance: 296.84  on 230  degrees of freedom
##   (18 observations deleted due to missingness)
## AIC: 300.84
##
## Number of Fisher Scoring iterations: 5
##
## Subject id:  8 , Coefficients, SE, and CI              Estimate Std. Error   z value     Pr(>|z|)
## (Intercept) -0.3332912  0.1543513 -2.159303 0.0308266818
## x            3.3351478  0.9044588  3.687451 0.0002265114
## Subject id:  8 , Null model comparison (Chi)Analysis of Deviance Table
##
## Model: binomial, link: logit
##
## Response: y
##
## Terms added sequentially (first to last)
##
##
##      Df Deviance Resid. Df Resid. Dev  Pr(>Chi)
## NULL                   231     321.55
## x     1   24.716       230     296.84 6.643e-07 ***
## ---
## Signif. codes:  0 '***' 0.001 '**' 0.01 '*' 0.05 '.' 0.1 ' ' 1
## Subject id:  9 , Logistic Regression Model Summary
## Call:
## glm(formula = y ~ x, family = "binomial", data = sub1)
##
```

```
## Deviance Residuals:
##     Min       1Q   Median       3Q      Max
## -1.9204  -0.8340  -0.7342   0.9220   1.7000
##
## Coefficients:
##             Estimate Std. Error z value Pr(>|z|)
## (Intercept)  -1.1762     0.2122  -5.544 2.96e-08 ***
## x             2.9605     0.4817   6.146 7.93e-10 ***
## ---
## Signif. codes:  0 '***' 0.001 '**' 0.01 '*' 0.05 '.' 0.1 ' ' 1
##
## (Dispersion parameter for binomial family taken to be 1)
##
##     Null deviance: 302.36  on 219  degrees of freedom
## Residual deviance: 257.08  on 218  degrees of freedom
##   (30 observations deleted due to missingness)
## AIC: 261.08
##
## Number of Fisher Scoring iterations: 4
##
## Subject id:  9 , Coefficients, SE, and CI             Estimate Std. Error   z value     Pr(>|z|)
## (Intercept) -1.176206  0.2121680 -5.543748 2.960644e-08
## x            2.960466  0.4816585  6.146400 7.926142e-10
## Subject id:  9 , Null model comparison (Chi)Analysis of Deviance Table
##
## Model: binomial, link: logit
##
## Response: y
##
## Terms added sequentially (first to last)
##
##
##      Df Deviance Resid. Df Resid. Dev  Pr(>Chi)
## NULL                   219     302.36
## x     1   45.284       218     257.08 1.705e-11 ***
## ---
## Signif. codes:  0 '***' 0.001 '**' 0.01 '*' 0.05 '.' 0.1 ' ' 1
## Subject id:  10 , Logistic Regression Model Summary
## Call:
## glm(formula = y ~ x, family = "binomial", data = sub1)
##
## Deviance Residuals:
##     Min       1Q   Median       3Q      Max
## -1.6801  -0.8251  -0.6811   0.9160   1.8218
##
## Coefficients:
##             Estimate Std. Error z value Pr(>|z|)
## (Intercept)  -1.4484     0.2252  -6.433 1.25e-10 ***
## x             2.6331     0.4915   5.357 8.45e-08 ***
## ---
## Signif. codes:  0 '***' 0.001 '**' 0.01 '*' 0.05 '.' 0.1 ' ' 1
##
## (Dispersion parameter for binomial family taken to be 1)
##
```

```
##     Null deviance: 287.27  on 219  degrees of freedom
## Residual deviance: 254.88  on 218  degrees of freedom
##   (30 observations deleted due to missingness)
## AIC: 258.88
##
## Number of Fisher Scoring iterations: 4
##
## Subject id:  10 , Coefficients, SE, and CI             Estimate Std. Error   z value     Pr(>|z|)
## (Intercept) -1.448416  0.2251694 -6.432560 1.254726e-10
## x            2.633064  0.4914897  5.357313 8.446868e-08
## Subject id:  10 , Null model comparison (Chi)Analysis of Deviance Table
##
## Model: binomial, link: logit
##
## Response: y
##
## Terms added sequentially (first to last)
##
##
##      Df Deviance Resid. Df Resid. Dev  Pr(>Chi)
## NULL                   219     287.27
## x     1   32.395       218     254.88 1.258e-08 ***
## ---
## Signif. codes:  0 '***' 0.001 '**' 0.01 '*' 0.05 '.' 0.1 ' ' 1
## Subject id:  11 , Logistic Regression Model Summary
## Call:
## glm(formula = y ~ x, family = "binomial", data = sub1)
##
## Deviance Residuals:
##      Min        1Q    Median        3Q       Max
## -2.59981   0.00003   0.00003   0.28166   1.60455
##
## Coefficients:
##             Estimate Std. Error z value Pr(>|z|)
## (Intercept)  -0.9643     0.6142   -1.57 0.116432
## x            22.9210     5.9692    3.84 0.000123 ***
## ---
## Signif. codes:  0 '***' 0.001 '**' 0.01 '*' 0.05 '.' 0.1 ' ' 1
##
## (Dispersion parameter for binomial family taken to be 1)
##
##     Null deviance: 119.71  on 219  degrees of freedom
## Residual deviance:  65.51  on 218  degrees of freedom
##   (30 observations deleted due to missingness)
## AIC: 69.51
##
## Number of Fisher Scoring iterations: 9
##
## Subject id:  11 , Coefficients, SE, and CI              Estimate Std. Error   z value     Pr(>|z|)
## (Intercept) -0.9642922  0.6142275 -1.569927 0.1164321348
## x           22.9209822  5.9692389  3.839850 0.0001231095
## Subject id:  11 , Null model comparison (Chi)Analysis of Deviance Table
##
## Model: binomial, link: logit
```

```
##
## Response: y
##
## Terms added sequentially (first to last)
##
##
##      Df Deviance Resid. Df Resid. Dev  Pr(>Chi)
## NULL                   219     119.70
## x     1   54.196       218      65.51 1.815e-13 ***
## ---
## Signif. codes:  0 '***' 0.001 '**' 0.01 '*' 0.05 '.' 0.1 ' ' 1
```
