# Supplementary material for: Validating a model of architectural hazard visibility with low-vision observers
Source: PLoS One. 2021 Nov 22;16(11):e0260267. doi: 10.1371/journal.pone.0260267 (PMC8608317; doi:10.1371/journal.pone.0260267)
Supplement: Attachment — Submitted filename: Consent Request Result 210920.pdf [file pone.0260267.s005.pdf]

Siyun Liu <liux4433@umn.edu>

Content Request Result for Liu et al. Manuscript (PONE-D-21-08870R1)

1 message

Siyun Liu <liux4433@umn.edu> Mon, Sep 20, 2021 at 8:00 AM
To: plosone@plos.org

Dear Editor,

Thanks for your attention! I'm the first author of the manuscript PONE-D-21-08870R1, Validating a model of architectural hazard visibility with low-vision observers. On 9/2, I received a message from PLOS One asking about the overlap between the submitted manuscript and a related paper (William B. Thompson, Robert A. Shakespeare, Siyun Liu, Sarah H. Creem-Regehr, Daniel J. Kersten & Gordon E. Legge (2021): Evaluating the Visibility of Architectural Features for People with Low Vision – A Quantitative Approach, *LEUKOS*, https://doi.org/10.1080/15502724.2021.1890115).

Here are our answers to the three questions.
1. Are any of the data of experiment 2 reproduced in any way in this submission?
- No. The data in experiment 2 were not reproduced in the current submitted manuscript.
2. Do any of Figures 1, 2, 7, and/or 10 appear in Thompson et al.?
- No. None of the figures in the current manuscript appeared in the Thompson et al. (2021) paper.
3. Do any of Figures 1, 2, 7, and/or 10 present data that were also presented in the Thompson et al. paper?
- Figure 7 presented data from Experiment 2, which was also included in the Thompson et al. (2021) paper.

We then contacted the Editor-in-chief of the LEUKOS journal, where the Thompson et al. (2021) paper was published, Kevin Houser. Our request message and his reply were both copied below. According to Dr. Houser, since Figure7 was not exactly identical with any figures published by LEUKOS, no copyright permission should be needed.

I hope the reply from Dr. Houser meets the clarification required by PLOS One. Please let me know if you need any other information. Thanks again for your time!

Best,
Siyun Liu

---------- Forwarded message ---------
From: **Kevin Houser** <khouser@ies.org>
Date: Thu, Sep 16, 2021 at 3:01 PM
Subject: RE: EXTERNAL : Content Copyright Request for Thompson et al. (2021)
To: Siyun Liu <liux4433@umn.edu>

Dear Siyun,

Copyright permission would be needed if your intent was to reproduce a figure (or table, etc.) exactly. The situation that you have described below does not require copyright permission. Thank you for checking.

Best wishes with the publication of this related work.

Sincerely,

Kevin

--

Kevin Houser, PhD, PE (NE), FIES, LC, LEED AP

Professor | School of Civil & Construction Engineering | Oregon State University

kevin.houser@oregonstate.edu

Chief Engineer | Pacific Northwest National Laboratory

https://www.pnnl.gov/advanced-lighting

Editor-in-Chief | LEUKOS, the journal of the Illuminating Engineering Society

khouser@ies.org | https://www.tandfonline.com/toc/ulks20/current

Pronouns: he, him, his

From: Siyun Liu <liux4433@umn.edu>
Sent: Tuesday, September 14, 2021 7:06 AM
To: Kevin Houser <khouser@ies.org>
Subject: EXTERNAL : Content Copyright Request for Thompson et al. (2021)

CAUTION: This email originated from outside of the organization. Do not click links or open attachments unless you recognize the sender and know the content is safe.

Dear Editor Houser,

Thanks for your attention!

I'm a coauthor of a paper published April 2021 in the LEUKOS journal, whose reference is:

William B. Thompson, Robert A. Shakespeare, Siyun Liu, Sarah H. Creem-Regehr, Daniel J. Kersten & Gordon E. Legge (2021): Evaluating the Visibility of Architectural Features for People with Low Vision – A Quantitative Approach, *LEUKOS*, https://doi.org/10.1080/15502724.2021.1890115

The LEUKOS number of this paper is 1890115.

Our LEUKOS paper mainly focused on describing the rationale, design, and implementation of the visibility evaluation software, with a section briefly mentioning a validation study of low-vision participants.

The extensive details of the validation study are included in a standalone manuscript, which is currently in review at the PLOS ONE journal. Its reference information being:

Liu S., Liu, Y., Kersten, D. J., Shakespeare, R. A., Thompson, W. B., & Legge, G. E. (in review) Validating a model of architectural hazard visibility with low-vision observers. *PLOS ONE.*

I am the first author of this manuscript. I am attaching a copy of the draft under review. It includes a more comprehensive treatment of the low-vision data, including additional analyses. It also includes data on normally sighted subjects not reported in the LEUKOS paper.

Figure 12 in our LEUKOS paper presented a summary of low-vision data as a logistic regression model fitted from the experiment data as log odds ratio plotted against the predictor variable. Figure 7 in our PLOS ONE article under review visualized the same summary data in a different format--as probability plotted against the predictor variable. The two figures have different formats but apply to the same summary data.

PLOS ONE is an open-access journal. The content they publish is shared under the CC BY 4.0 license, which means allowing unrestricted use and distribution, even commercially, by third parties. The PLOS ONE journal wants to make sure the relevant copyright holder gives the consent. Due to the content overlap between these two papers, I wish to request the permission from you that the experiment result presented in the Thompson et al. (2021) can also be published by PLOS ONE in the Liu et al. (in review) manuscript.

Here is the statement of request recommended by PLOS ONE:

I request permission for the open-access journal PLOS ONE to publish

Liu S., Liu, Y., Kersten, D. J., Shakespeare, R. A., Thompson, W. B., & Legge, G. E. (in review) “Validating a model of architectural hazard visibility with low-vision observers”. *PLOS ONE*

under the Creative Commons Attribution License (CCAL) CC BY 4.0 (http://creativecommons.org/licenses/by/4.0/). Please be aware that this license allows unrestricted use and distribution, even commercially, by third parties. Please reply and provide explicit written permission to publish

Liu S., Liu, Y., Kersten, D. J., Shakespeare, R. A., Thompson, W. B., & Legge, G. E. (in review) Validating a model of architectural hazard visibility with low-vision observers. *PLOS ONE*

under a CC BY license.

I am attaching a PLOS ONE Request for Permission form. I sincerely wish you would consider my request and give your signature on the permission form. If you would give your permission, please kindly fill in the permission form. If you have any concerns, please feel free to contact me with your questions.

Best regards,

Siyun Liu

--

Siyun Liu, Ph.D. Student,

Motion Perception and Low Vision Navigation

MN Lab for Low Vision Research, University of Minnesota

N37 Elliot Hall, 75 East River Drive, Minneapolis, MN

liux4433@umn.edu

--

Siyun Liu, Ph.D. Student,
Motion Perception and Low Vision Navigation
MN Lab for Low Vision Research, University of Minnesota
N37 Elliot Hall, 75 East River Drive, Minneapolis, MN
liux4433@umn.edu
